# Purpura fulminans secondary to *Capnocytophaga canimorsus* bacteraemia following a dog bite: A case report and review of literature

**DOI:** 10.1099/acmi.0.000505.v3

**Published:** 2023-06-16

**Authors:** Kieran Killington, Nathaniel Lee, Radha Asher, Olivia Farrant, Neil Stone

**Affiliations:** ^1^​ University College Hospital London, 235 Euston Road, N1 2BU, UK; ^2^​ Hospital Of Tropical Diseases, Mortimer Market, Capper St, London WC1E 6JB, UK

**Keywords:** *Capnocytophaga canimorsus*, purpura fulminans, antimicrobial resistance

## Abstract

**Introduction.:**

Infection due to *Capnocytophaga canimorsus* may result in a wide variety of clinical presentations. We present a case of life-threatening *Capnocytophaga canimorsus* infection with evolution of ecchymosis to purpura fulminans.

**Case description.:**

We present a case of a 43-year-old male with a history of excessive alcohol consumption who presented with features of sepsis following a dog bite. This was associated with a striking, widespread purpuric rash. A causative pathogen, *C. canimorsus* was identified through blood culture and 16S RNA sequencing. His initially purpuric rash underwent bullous transformation and was diagnosed clinically as purpura fulminans, confirmed on skin biopsy. He made a full recovery with prompt antimicrobial therapy, initially with co-amoxiclav but escalated to clindamycin and meropenem due to clinical deterioration and concerns of beta-lactamase resistance.

**Discussion.:**

β-Lactamase producing *Capnocytophaga* strains are of increasing concern. This particular concern is reflected in our case as 5 days into treatment with β-lactamase inhibitor combination therapy the patients clinical condition deteriorated but demonstrably improved on switching to a carbapenem.

The development of biopsy proven purpura fulminans in this immunocompetent case is a rare severe manifestation of the previously reported manifestation of disseminated intravascular coagulation (DIC) in *Capnocytophaga* bacteraemia. The case reported describes characteristics common with other DIC presentations such as the presence of clinical risk factors (history of excessive alcohol consumption) and symmetrical involvement. However, an unusual feature in that initial purpuric lesions were followed by the development of a bullous appearance and peripheral necrotic features concerning for purpura fulminans and confirmed with skin biopsy.

## Data Summary

No data was generated in the production of this article.

## Introduction

*Capnocytophaga canimorsus* is a facultative anaerobic, capnophilic, Gram-negative bacillus which is typically found in the oral flora of dogs. Owing to its slow growth, it was first described as dysgonic fermenter-2 in 1976 but renamed in 1989 to *Capnocytophaga canimorsus* due to its association with dog bites. *C. canimorsus* takes up to 14 days to culture, which can result in false negative blood culture results [1, 2], and presents a diagnostic challenge.

Infection is often due to a dog bite but may be through licking, biting or scratching. Dogs contain many pathogenic bacteria in their oropharynx, including *Pasteurella canis *, *Streptococcus* spp., *Staphylococcus* spp., *Fusobacterium* spp., *Bacteroides* spp., and *Capnocytophaga canimorsus* [3]. Most cases of *C. canimorsus* sepsis are related to an immune disorder such as splenectomy, chronic alcohol use, or immunodeficiency, but no identifiable risk factor has been found in 40 % of cases [2].

Infection due to *C. canimorsus* may result in a wide variety of clinical presentations; sepsis being the most common and most serious, with mortality rates in septic patients of up to 55 % [1]. A wide range of clinical presentations can occur, including meningitis, intra-abdominal infection, endocarditis, infectious arthritis, brain abscess, endophthalmitis and pneumonia. Cutaneous signs are common, often petechial rashes or ecchymosis, and can progress to purpura fulminans or gangrene requiring amputation [2]. Purpura fulminans (PF) is a rare, life-threatening complication of bacterial sepsis which occurs due to a pro-thrombotic subtype of disseminated intravascular coagulation (DIC). Patients with PF develop protein C (PC) deficiency, a protease which acts as an endogenous anticoagulant [4]. Bleeding disorders, including DIC, are common in *C. canimorsus* sepsis, and can result in purpura fulminans or gangrene.

Treatment of *C. canimorsus* is recommended with a carbapenem, clindamycin or beta-lactamase inhibitor combinations [1] such as co-amoxiclav, due to reports of beta-lactamase resistant *C. canimorsus* [2]. We present a case of a patient presenting with features of *Capnocytophaga canimorsus* sepsis with evolution of ecchymosis to purpura fulminans, evidenced by skin biopsy.

## Case description

A 43-year-old male with a significant history of alcohol use presented with a 24 h history of a painful rash across his torso, back, arms and legs. He had been vomiting in the 48 h prior, described cold peripheries with difficulty walking, and reported daily heroin, crack cocaine and alcohol use. He reported a chronic long-standing cough, with no shortness of breath, chest pain or haemoptysis. He also reported being bitten by a friend’s dog a week prior to admission. He was born in the UK and had always lived in London.

**Fig. 1.**
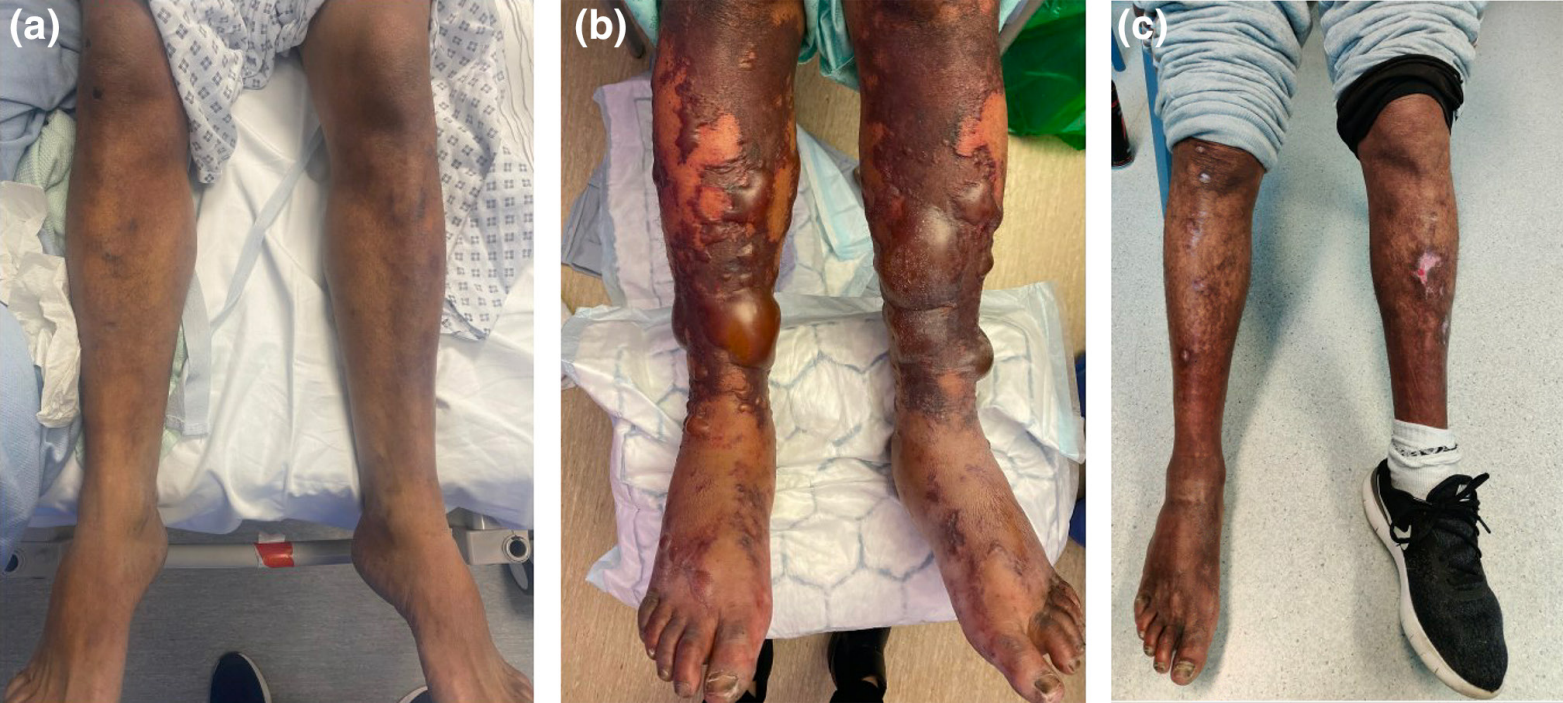
(a) On admission (b) bullous ‘transformation’ (c) resolution of rash.

On examination, he was tachycardic (HR 135), with cool peripheries with a non-blanching widespread purpuric rash (Fig. 1a). He had reduced sensation and power in the lower limbs bilaterally. He had epigastric tenderness on abdominal examination. He had no features of deep vein thrombosis.

Admission blood tests are shown in Table 1.

**Table:** T**able** 1. Admission blood tests

Marker		
White cell count	15	×10^9^
Neutrophils	14	×10^9^
C-reactive protein	503	mg l^−1^
Sodium	129	Mmol l^−1^
Creatinine	161	Umol l^−1^
Urea	14	Mmol l^−1^
ALT	761	IU l^−1^
ALP	133	IU l^−1^
Bilirubin	38	Umol l^−1^

Given the broad symptomatology and multiple blood test derangements, he was extensively investigated. Chest X-ray showed diffuse reticular opacity bilaterally particularly in the mid and lower zones, which was similar in appearance to a previous radiograph in 2017. CT pulmonary angiography, requested for raised d-dimer and sinus tachycardia, was negative for pulmonary emboli. Stool samples were collected and sent for bacterial and viral PCR, plus ova, cysts and parasites. Sputum microscopy was requested to exclude chest infection. Furthermore, due to hyponatraemia and abnormal liver function tests, serology was tested for mycoplasma antibodies, and urine for pneumococcal and legionella antigens. A blood borne viral screen (HIV, hepatitis B and C) and full vasculitis screen was sent. Two sets of blood cultures were taken before starting co-amoxiclav for broad coverage of sepsis of unknown origin.

After reviewing fibrinogen (3.9 g l^−1^), LDH (2234 IU l^−1^), and haptoglobins (0.7 g l^−1^), haematology felt that the presentation was likely platelet consumption in the context of inflammation or infection. His urine dip was positive for blood and protein, so streptococcus titre, throat swab and microscopy and culture were further requested for consideration of haemolytic uraemic syndrome given evidence of nephritic syndrome, infection and deranged clotting function. At this time, his rash was becoming more widespread and felt to be most likely vasculitic in nature. His vasculitic screen was negative for ANA, ANCA, ENA, Jo-1, LA, RNP, RO, Scl-70, SM, anti-Scl RF was mildly elevated at 26 IU ml^−1^. C3 was 0.93 g l^−1^, C4 0.44 g l^−1^. ASO titre was negative. His HIV, hepatitis B and C, COVID status and syphilis were negative. His legionella, mycoplasma, and pneumococcal antigens were negative. Full respiratory viral swab was negative. As asplenism is a risk factor for *Capnocytophaga* infection, an ultrasound of the spleen was requested, which was normal. The blood film was reported as demonstrating toxic neutrophil changes, in keeping with infection.

Two days into his admission, his blood culture was positive for Gram-negative rods in the anaerobic bottle. Following microbiology multidisciplinary team (MDT) discussion, 16S PCR and a further blood sample to assess for bacteria in the buffy coat was sent, as *Capnocytophaga* infection was increasingly likely. Despite an initial improvement on day one of admission, the patients white cell count (WCC) had increased to 21.3×10^9^ l^−1^ (15.4×10^9^ l^−1^), Nt 16.5×10^9^ l^−1^ (11.3×10^9^ l^−1^) with a static C-reactive protein (CRP) at 171 mg l^−1^ (179 mg l^−1^) with ongoing low grade temperature spikes. Due to reports of beta-lactamase resistance in *Capnocytophaga *, clindamycin was added (600 mg IV TDS) empirically. A further blood culture was returned positive for Gram-negative rods in the anaerobic bottle on day 3. His platelet count decreased to nine, for which haematology advised testing for ADAMTS13 to exclude thrombotic thrombocytopenic purpura. vWF cleaving protease levels were low (51.8 IU dl^−1^), with raised vWF antigen (7.52 IU ml^−1^).

On the fifth day of his admission, the rash underwent bullous ‘transformation’ (Fig. 1b) on the limbs and flanks. The rash was exquisitely tender and two lesions on the knee were annular in appearance with some necrotic changes. A skin biopsy was taken for histopathology, culture and immunofluorescence. Purpura fulminans secondary to *Capnocytophaga* bacteraemia was likely, but due to static CRP (175 mg l^−1^ [171 mg l^−1^]) with worsening white cell count (WCC 24.8×10^9^ l^−1^ [21.3×10^9^ l^−1^], Nt 19.1×10^9^ l^−1^ [16.5×10^^9^ l^−1^]) an underlying necrotising fasciitis was considered. An urgent surgical opinion was requested and antibiotic cover was broadened to meropenem with clindamycin, and a stat dose of amikacin was given. The patient had further fevers, but the rash had not progressed by morning, so it was felt to not be in keeping with necrotising fasciitis.

The following day, the patient began to improve clinically, with observations returning to normal parameters, inflammatory markers were down-trending, and his pain was well controlled, allowing him to mobilise with the assistance of two crutches. His improvement continued over the course of 5 days, where his inflammatory markers had decreased (WCC 7.9×10^9^ l^−1^, Nt 4.0×10^9^ l^−1^, CRP was 107 mg l^−1^) resulting in change of antibiotics to oral co-amoxiclav and clindamycin. He was educated on how to dress his wound ulcers and was discharged after a thirteen-day admission with Infectious Diseases clinic follow-up. The antibiotics were continued at follow-up clinic for a total of 5 weeks, where the rash had resolved (Fig. 1c). Sixteen days after discharge, the 16S sequencing confirmed *Capnocytophaga canimorsus* 16S rDNA. Susceptibility testing was not possible as the organisms did not grow on culture. Punch biopsy results were later returned, showing:

MACROSCOPIC DESCRIPTION: Skin biopsy punch right lower leg: tan punch biopsy measuring 4×4 mm and up to a depth of 5 mm. The skin surface looks crusty and peeling off probably bullae peeling off.

**Fig. 2.**
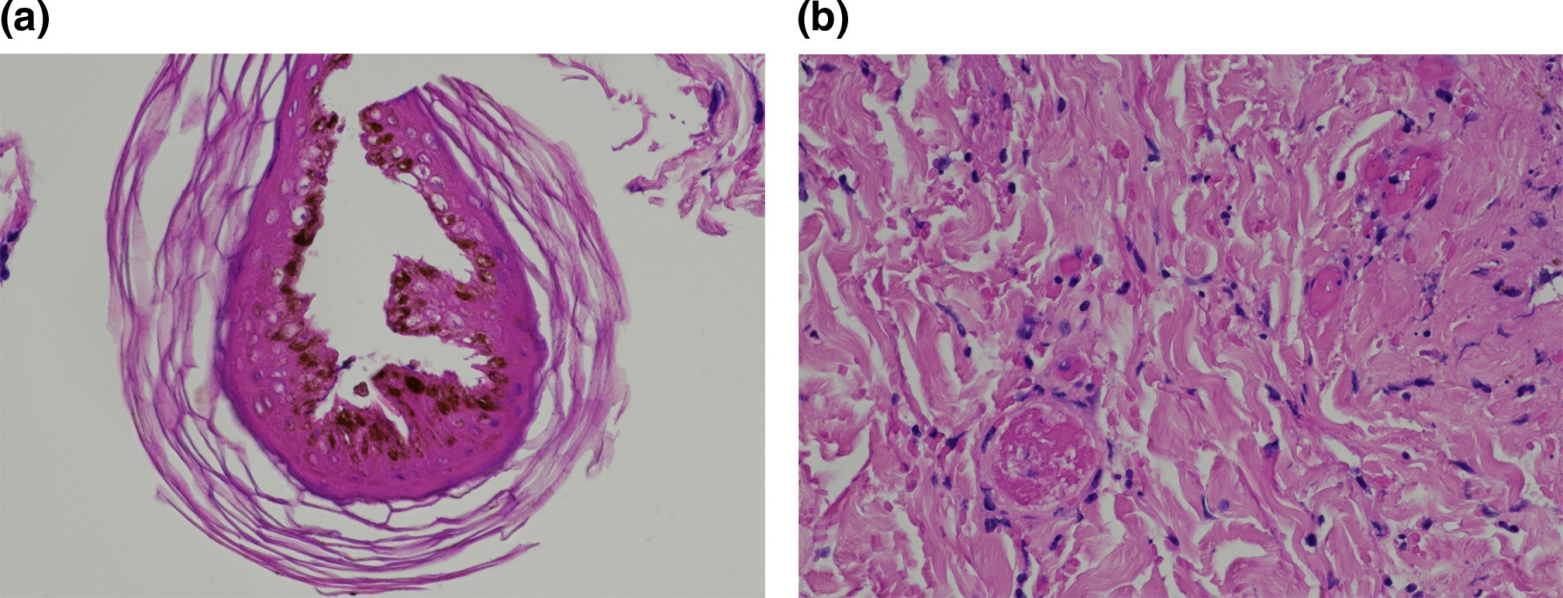
(a) A detached fragment of necrotic epidermis (b) thrombi with dermal capillaries and adjacent erythrocyte extravasation.

MICROSCOPIC DESCRIPTION: Section shows a skin punch biopsy including detached fragments of necrotic epidermis and ulcer slough. There is thrombotic occlusion of small dermal vessels with erythrocyte extravasation, a neutrophilic infiltrate and necrosis of eccrine glands and hair follicles. The morphological features favour ischaemic necrosis on a background of thrombotic vasculopathy. (Fig. 2a, b)

## Discussion

In this report, we describe an unusual manifestation of rare infection caused by a dog bite. Skin manifestations are common in cases of sepsis caused by *C. canimorsus *, however progression to purpura fulminans is rare. We present pictures showing the evolution of the dermatological findings, as well as a skin biopsy, which is often excluded due to severe coagulopathy. We also show resolution rash through follow-up appointments. Table 2 outlines the reported cases in the literature of *Capnocytophaga canimorsus* infection associated with purpura fulminans, of which we found 20 reported cases. Common themes in all cases are long bacterial culture time (max 11d), use of broad-spectrum antibiotics, and severity of infection – with 11/20 cases managed on intensive care units, and 7/20 cases resulting in death.

**Table 2.** Summary of case reports of *Capnocytophaga canimorsus* associated with purpura fulminans. Where values are blank in the table, they were not described in the paper

	Kullberg *et al*. (1991) [16]	MS. Morgan (1994) [17]	Bryson *et al*. (2003) [18]	Gonzales *et al*. (2004) [19]	Deshmukh *et al*. (2004) [20]
**Age/Sex**	35F	37M	49F	59M	45M
**Exposure**	Dog bite	Dog bite	Dog bite	Dog contact (suspected lick)	Dog bite
**Risk factors**	Nil noted	Splenectomy (Hogkin lymphoma)	Nil noted	Nil noted	Nil noted
**Symptoms/ signs on admission**	Abdominal pain Headache Myalgia Fever	Malar purpura Gangrene of tip of nose	Flu-like symptoms	Febrile Bilateral purpuric rash affecting feet and pretibial areas	Lower extremity pain Hoarseness Dyspnoea Generalised cyanotic mottling of the skin
***Investigations***					
**Hb**	8.4 mmol l^−1^		13.1 g dl^−1^	9.6 g dl^−1^	
**WCC (Nt**)	15.3×10^9^ l^−1^		1.7×10^9^ l^−1^	18.6 ul^−1^	13300 cells mm^−3^
**Platelets**	12.0×10^9^ l^−1^		23×10^9^ l^−1^	21.8 ul^−1^	24000 cells mm^−3^
**Creatinine**	419 umol l^−1^			2.4 mg dl^−1^	2 mg dl^−1^
**INR/PT/APTT**	PT 22.8 APTT 86 s		PT 24 APTT 58 s	INR 2	PT 17.7 INR 2.25
**CRP**					
**Diagnosis**	AKI DIC Purpura fulminans	DIC Comatose for 36 days, unclear cause Purpura fulminans	AKI requiring haemodialysis Purpura fulminans	Purpura fulminans AKI Respiratory failure (required mechanical ventilation)	Purpura fulminans DIC Metabolic acidosis (requiring dialysis) Respiratory failure
**Identification**	Culture (8d)	Culture (11d)	Culture	Culture (72 h)	Culture
**Treatment**	Required intensive care (ITU) admission Penicillin G Cloxacillin Ceftazidime	Benzylpenicillin Cefotaxime Flucloxacillin Metronidazole, changed to just cipro on day 11	Antibiotics FFP	Co-amoxiclav, piperacillin-tazobactam, then switched to Imipenem 1 g QDS 10/7 course when no improvement	Intubation Haemodialysis IV imipenem and azithromycin with doxycycline
**Outcome**	Survived	Survived	Died (4d)	Survived	Died (7d)
	**Völl *et al*. (2007) [21]**	**Christiansen *et al*. (2012) [22]**	**Christiansen *et al*. (2012) [22]**	**Tay *et al*. (2012) [23]**	**Kennel *et al*. (2014) [24]**
**Age/Sex**	61M	59F	59M	60F	50M
**Exposure**	Dog bite	Dog bite	Dog contact (lick)	Dog bite	Dog contact
**Risk factors**	Nil noted	Nil noted	Nil noted	Splenectomy (TTP)	Nil noted
**Symptoms/signs on admission**	Back pain Abdominal pain	Nausea Vomiting Diarrhoea Widespread ecchymoses	Fever Abdominal pain Diarrhoea	Purpura fulminans DIC Multiorgan failure	Abdominal pain Nausea Vomiting Fever
***Investigations***					
**Hb**		5.4 mmol l^−1^	6.9 mmol l^−1^	113 g l^−1^	
**WCC (Nt**)		18.1×10^9^ l^−1^	0.8×10^9^ l^−1^	11.3 (8.3)	
**Platelets**		16×10^9^ l^−1^	16×10^9^ l^−1^	38×10^9^ l^−1^	
**Creatinine**		278 umol l^−1^	278 umol l^−1^		
**INR/PT/APTT**		INR1.6 APTT 67 s	INR2.3 APTT 180 s		
**CRP**		2071 nmol l^−1^	1942 nmol l^−1^		
**Diagnosis**	Purpura fulminans	Gangrene of fourth finger DIC Purpura fulminans	DIC Haemolytic anaemia Renal failure Multiple myeloma Gangrene all toes Purpura fulminans	Gangrene of hand and toes Purpura fulminans	Purpura fulminans affecting the face
**Identification (time taken**)	Culture +gene sequencing	Bacterial culture (8d)	Bacterial culture (5d)	Bacterial culture	Not described
**Treatment**	piperacillin/tazobactam and hydrocortisone for septic shock	Empiric clindamycin, ciprofloxacin and clarithromycin	Required ITU admission Cefuroxime, ciprofloxacin, metronidazole	Broad spectrum Abx, ITU admission (no specific mention of inotropes / vasopressors)	Ceftriaxone and ofloxacin (co-amoxiclav allergy)
**Outcome**	Died (4d)	Survived	Survived	Died	Survived
	**Sotiriou *et al*. (2015) [25]**	**Bertin *et al*. (2018) [26]**	**Mantovani *et al*. (2018) [27]**	**Bendapudi *et al*. (2018) [4]**	**Harana *et al*. (2019) [28]**
**Age/Sex**	57M	69F	80F	39M	50F
**Exposure**	Dog bite	Dog bite	Dog bite	Dog bite	Dog bite
**Risk factors**	Nil noted	Nil noted	Nil noted	Nil noted	Nil noted
**Symptoms/signs on admission**	Tachypnoea Abdominal pain Fever Widespread livedo reticularis	SOB Fatigue Fever Sepsis	Confusion Fever Abdominal pain	Sore throat Malaise Purpuric rash	Septic shock Acral necrosis Dry gangrene
***Investigations***					
**Hb**				14.2 g dl^−1^	
**WCC (Nt**)	21×10^9^ l^−1^			6180 mm^−3^	
**Platelets**	18×10^9^ l^−1^	22 mm^−3^		6 mm^−3^	
**Creatinine**	2.19 mg dl^−1^	4 mg dl^−1^		5.10 mg dl^−1^	
**INR/PT/APTT**		INR 1.9	INT 2.79	INR 8.9 APTT >150 s	
**CRP**		29 mg dl^−1^	6.1 mg dl^−1^		
**Diagnosis**	Purpura fulminans with ecchymosis DIC Multiorgan failure	Renal and respiratory failure Gangrene of hands and toes DIC Ischaemic CVA	Purpura fulminans	Purpura fulminans	DIC Distal gangrene
**Identification (time taken**)	MALDI-TOF	Molecular techniques	Bacterial culture	PCR assay	Not described
**Treatment**	Required ITU admission	Required ITU admission, Piperacillin-tazobactam, vancomycin	Piperacillin-tazobactam (18 g/24 h infusion)	Required ITU admission PC concentrate Antibiotics	Given vasopressors and inotropes Surgical debridement No mention of Abx
**Outcome**	Survived	Survived	Died	Survived	Survived
	**Mader *et al*. (2019) [29]**	**Igeta *et al*. (2020) [30]**	**Terashima *et al*. (2020) [31]**	**Martins-Baltar *et al*. (2022) [32]**	**Martins-Baltar *et al*. (2022) [32]**
**Age/Sex**	63M	38M	58M	53M	38M
**Exposure**	Dog contact (lick)	Dog contact (lick)	Dog contact	Dog bite	Dog bite
**Risk factors**	Nil noted	Alcohol dependency	Nil noted	Alcohol consumption ‘without cirrhosis’	Nil noted
**Symptoms/signs on admission**	Fever Dyspnoea Petechiae	Fever Diarrhoea	Fever Reduced consciousness	Fever Diarrhoea Abdominal pain	Confusion Emesis Abdominal pain
***Investigations***					
**Hb**			14.3 g dl^−1^		
**WCC (Nt**)	0.2×10^3^ µl^−1^	16700 µl^−1^	3830 µl^−1^	3.96 G l^−1^	20 G l^−1^
**Platelets**	20×10^3^ µl^−1^	13000 µl^−1^	51000 µl^−1^	27 G l^−1^	7 G l^−1^
**Creatinine**	3.4 mg dl^−1^	2.08 mg dl^−1^	1.59 mg dl^−1^	149 µmol l^−1^	354 µmol l^−1^
**INR/PT/APTT**	PT >180 s	INR 2.32	INR 1.47, ATPP 51.7 s		
**CRP**	205 mg l^−1^		13.73 mg dl^−1^		
**Diagnosis**	Encephalopathy Paralytic ileus Acute renal and liver failure Pulmonary aspergillosis Purpura fulminans	DIC Compartment syndrome Purpura fulminans	DIC Septic shock HLH	Septic cardiomyopathy (LVEF <20 %) Purpura fulminans	Septic shock Purpura fulminans
**Identification (time taken**)	Culture (4d)	Culture (3d)	PCR Culture (10d)	16S RNA PCR Culture negative 48 h	16S RNA PCR (36 h) Culture (5d)
**Treatment**	Clarithromycin and piperacillin-tazobactam	Required ITU admission Meropenem 1,000 mg IV q8h, vancomycin 1,250 mg IV q12h, minocycline 100 mg IV q12 h, tobramycin 240 mg IV OD)	Meropenem and methylprednisolone 1 g/day	Empirical ceftriaxone, clindamycin and metronidazole. Switched to ceftriaxone monotherapy for 14d after positive PCR	Intubation Haemodialysis empirical antibiotic therapy with ceftriaxone, gentamicin and metronidazole Ceftriaxone 10 days
**Outcome**	Died (16d)	Died	Survived	Survived	Survived

Purpura fulminans is a pro-thrombotic subtype of DIC characterised by intravascular thrombosis and haemorrhagic infarction of the skin. DIC is a clinicopathological syndrome, requiring both clinical and biochemical markers for diagnosis. This patient presented with reduced platelet count, raised d-dimer, and prolonged APTT in the context of bacterial sepsis. The fibrinogen in this case was normal, which is seen in up to 57 % of patients with DIC [5]. The International Society on Thrombosis and Haemostasis (ISTH) has created the ISTH score to aid DIC diagnosis. In this case, the patient meets the diagnostic criteria for DIC, and has findings consistent with PF on skin biopsy [6]. Treatment is with supportive care, with some evidence that administration of protein C concentrate is associated with reversal of progressive ischaemia and improvement of laboratory values associated with DIC. Study sizes however are small (*n=*12), with even smaller samples looking at *C. canimorsus* infection (*n=*2) [7]. Whilst protein C deficiency plays a role in the pathophysiology of purpura fulminans, *Capnocytophaga* species cause DIC through production of a proteolytic enzyme (type seven dipeptidyl peptidase) which acts as a local inhibitor of factor-X mediated coagulation, and so this benefit of protein C replacement may be limited [8].

Diagnosis of *Capnocytophaga* infection is complicated by the specific conditions required for culture [9], and the longer incubation time (up to 14 days). This highlights the need for a thorough history and examination, as prompt administration of an appropriate antibiotic is associated with better outcomes in sepsis. The microbiology lab should be alerted if *Capnocytophaga* is suspected, as routine cultures are typically discarded after 5 days. The 16S RNA PCR sequencing is increasingly used in diagnosis of *Capnocytophaga* infections, as it yields quicker results than traditional culture techniques, and has shown better outcomes in culture-negative infections [9]. Indeed, 16S RNA PCR was required for diagnosis in this case, and sensitives were never revealed due to organisms not surviving culture.

The fastidious growth of *C. canimorsus* often results in empirical antibiotic use, which is of particular concern given reports of resistant *Capnocytophaga* species. Beta-lactamase producing *C. canimorsus* have been identified and should be considered in any case of poor response to beta-lactamase combination therapy. This is highlighted in similar literature (Table 2), where broad spectrum antimicrobials, often in combination, are commonly used. *Capnocytophaga* spp. are typically sensitive to clindamycin, linezolid, tetracycline, chloramphenicol, imipenem and beta-lactamase inhibitor combinations. Due to increasing number of beta-lactamase-producing species, treatment is generally recommended with clindamycin or beta-lactamase inhibitor combination drugs. Both chromosomal and plasmid-encoded beta-lactamases have been identified of Ambler classification class A group 2e, including *bla*Cfx and *bla*CSP-1 [10]. These genes encode extended-spectrum beta-lactamases (ESBLs), the activity of which is inhibited by clavulanic acid. In beta-lactamase producing *Capnocytophaga *, *bla*CSP has been identified in over 60%, and *bla*Cfx in 30–80 % of isolates [11, 12]. The wide range is likely due to geographical diversity. Mutations in DNA gyrase (*gyrA*) have been implicated in fluoroquinolone resistance [13]. Multi-drug resistant isolates requiring treatment with carbapenems have been reported, although the genomic organisation of *Capnocytophaga* was not elucidated in this case [14]. Whole genome sequencing of *Capnocytophaga* spp. has revealed presence of Class D beta-lactamase genes (blaOXA-347), associated with phenotypic resistance to penicillin, cephalosporin, and to imipenem [15]. Notably, these isolates were found on the bacterial chromosome of non-*canimorsus* strains, and only in wound, and not blood, culture samples. This suggests resistance may be associated with a fitness cost to virulence, and as blaOXA-347 is found in chromosomal DNA, reduced risk of transmission from strain to strain. Given the worldwide popularity of pet ownership, a OneHealth approach to investigating mobile genetic elements for antimicrobial resistance in canine oral flora, would inform the future risk of MDR infections from dog contact. Further genome sequencing and genotype-phenotype correlation is required to understand antimicrobial resistance in *Capnocytophaga* species, which may lead to quicker detection that culture and routine sensitivity testing.
